# A Pea (*Pisum sativum* L.) Seed Vicilins Hydrolysate Exhibits PPARγ Ligand Activity and Modulates Adipocyte Differentiation in a 3T3-L1 Cell Culture Model

**DOI:** 10.3390/foods9060793

**Published:** 2020-06-16

**Authors:** Raquel Ruiz, Raquel Olías, Alfonso Clemente, Luis A. Rubio

**Affiliations:** Physiology and Biochemistry of Animal Nutrition, Estación Experimental del Zaidín (EEZ, CSIC), 18008 Granada, Spain; rruiz@eez.csic.es (R.R.); raquel.olias@eez.csic.es (R.O.); alfonso.clemente@eez.csic.es (A.C.)

**Keywords:** adipocyte, hydrolysate, *Pisum sativum*, PPARγ, vicilin

## Abstract

Legume consumption has been reported to induce beneficial effects on obesity-associated metabolic disorders, but the underlying mechanisms have not been fully clarified. In the current work, pea (*Pisum sativum* L.) seed meal proteins (albumins, legumins and vicilins) were isolated, submitted to a simulated gastrointestinal digestion, and the effects of their hydrolysates (pea albumins hydrolysates (PAH), pea legumins hydrolysates (PLH) and pea vicilin hydrolysates (PVH), respectively) on 3T3-L1 murine pre-adipocytes were investigated. The pea vicilin hydrolysate (PVH), but not native pea vicilins, increased lipid accumulation during adipocyte differentiation. PVH also increased the mRNA expression levels of the adipocyte fatty acid-binding protein (aP2) and decreased that of pre-adipocyte factor-1 (Pref-1) (a pre-adipocyte marker gene), suggesting that PVH promotes adipocyte differentiation. Moreover, PVH induced adiponectin and insulin-responsive glucose transporter 4 (GLUT4) and stimulated glucose uptake. The expression levels of peroxisome proliferator-activated receptor γ (PPARγ), a key regulator of adipocyte differentiation, were up-regulated in 3T3-L1 cells treated with PVH during adipocyte differentiation. Finally, PVH exhibited PPARγ ligand activity. Lactalbumin or other pea hydrolysates (PAH, PLH) did not exhibit such effects. These findings show that PVH stimulates adipocyte differentiation via, at least in part, the up-regulation of PPARγ expression levels and ligand activity. These effects of PVH might be relevant in the context of the beneficial health effects of legume consumption in obesity-associated metabolic disorders.

## 1. Introduction

Overweight and obesity are defined as abnormal or excessive fat accumulation that presents a risk to health. According to the World Health Organization WHO, overweight and obesity are regarded as world epidemics and are major risk factors for a number of chronic diseases, including diabetes, cardiovascular diseases and cancer. Obesity is associated with the Western diet [1], as eating patterns in Western industrialized countries are characterized by high energy consumption and chronic over-consumption of saturated fat, cholesterol, sugar and salt, which is also related to the development of other pathologies such as diabetes, cardiovascular and degenerative disorders, cancer, hepatic steatosis and obesity, that are hallmarks of the metabolic syndrome [2,3,4]. In this context, it has been proposed that weight loss could be achieved by consuming controlled energy diets with a high content of cereals and legumes [5]. Legumes have long been an important component of the human diet because of their content in protein, carbohydrates (mainly in the form of starch) and many other nutrients [6]. In addition, the substitution of energy-rich foods for pulses has been reported to have beneficial effects on the prevention and treatment of obesity and related disorders such as cardiovascular diseases, type 2 diabetes and the metabolic syndrome [7]. Several nutritional intervention studies confirm that the inclusion of legumes in the diet lowers the risk of developing overweight or obesity in adults [8,9,10], making them appropriate candidates for healthy slimming diets.

As indicated above, it is generally recognized that grain legumes not only contribute effectively to a well-balanced diet but can also prevent non-communicable diseases, including type II diabetes and cardiovascular diseases [11,12]. Both albumins and globulins (11S and 7S storage proteins) from legume seeds have been claimed to induce a number of health beneficial effects (hypoglycemic, hypocholesterolemic, antihypertensive, antiinflammatory, anticarcinogenic, etc.) upon dietary consumption [13], although the putative molecular mechanisms of action underlying those effects are still not properly clarified. However, it seems clear that in order to be properly evaluated, well-defined seed protein fractions have to be extracted and purified for appropriate testing. In a previous work [14] focused on chemical characterization of pea (*Pisum sativum* cv. Bilbo) seed protein fractions, we were able to obtain and characterize protein isolates (albumins, legumins and vicilins) with very low (or devoid of) cross contamination with other proteins. These isolates are therefore potentially useful to study the biological effects of native or treated pea proteins. For example, we have recently reported the preventive anti-inflammatory effect of a pea albumin isolate in a DSS (dextran sodium sulfate)-treated mice model [15,16].

Observations have been accumulating over the last decades to suggest that legume proteins, and particularly storage proteins (legumins and vicilins), are not only valuable sources of amino acids, but might also induce some physiological effects in animals or humans fed legume meal- or legume protein-based diets. Those effects may be classified into four main groups: (1) adverse immune reactions; (2) modulation of plasma lipids and cholesterol values; (3) increased intestinal excretion of endogenous proteins; (4) increased excretion of N through the urine, and changes in plasma amino acids concentrations [17]. Although still controversial, evidence for the absorption of significant amounts of peptides and even intact proteins during the normal digestive process has been reported in the literature [18,19,20]. Therefore, it does not seem unlikely that some absorbed peptide(s) arising from protein digestion in the intestine might be biologically active and produce certain metabolic effects [21,22,23]. In particular, Lovati et al. [24] found that soybean 7S and 11S globulins (legumins and vicilins, respectively) were able to increase the uptake and/or degradation of 125I-LDL in Hep G2 and HSF cell lines in vitro. In an in vitro model resembling the intestinal enterocyte layer (bicameral Caco-2 cell cultures), amino acids from legume protein hydrolysates were found to be absorbed at rates different from those of other proteins of animal origin such as casein [25], which is likely to be linked to differences in the protein primary structure of the different proteins. It seems therefore reasonable to hypothesize that differences in the absorption rate and/or chemical form (free amino acids, peptides) may be linked to the observed effects of legume proteins on lipids metabolism [26].

Goto et al., (2013) [27] showed previously that a soy protein hydrolysate induced beneficial effects on obesity metabolic disorders by promoting adipocyte differentiation. Accordingly, in the work here described we have investigated the effects of digested pea protein fractions on adipocyte differentiation by using 3T3-L1 murine pre-adipocytes. Rosiglitazone was used as positive control because thiazolidinediones are insulin sensitizers that promote the differentiation of pre-adipocytes by peroxisome proliferator-activated receptor γ (PPARγ) activation [28], which leads to an increase in the number of small adipocytes and to a decrease in the number of large adipocytes by increasing apoptosis [29]. Lactalbumin hydrolysate (Lactalb. H) was included for comparison with a protein of animal origin because it is commonly used together with casein as control protein in in vivo rat studies. Our main finding was that the pea vicilin hydrolysate (PVH) exhibited PPARγ ligand activity, significantly up-regulated PPARγ expression levels and induced adipocyte differentiation, leading to the enhancement of adiponectin production and insulin-stimulated glucose uptake. These effects of PVH have not been described previously to our knowledge for any legume protein apart from soybean hydrolysates, and might be relevant to understand the effects of pulse consumption on health, and particularly on obesity-associated metabolic disorders.

## 2. Materials and Methods

### 2.1. Chemicals

All chemicals were obtained from Sigma Chemical (Madrid, Spain), unless otherwise stated.

### 2.2. Pea Seed Fractionation Procedure

The procedure to obtain pea protein fractions has been previously described [14]. Briefly, pea (*Pisum sativum* L. cv. Bilbo) seeds were ground; the obtained meal was treated with chloroform: methanol for lipid extraction and air-dried. Defatted meal was extracted (1:10, *w:v*) with 0.2 M borate buffer pH 8.0 containing 0.5 M NaCl and centrifuged. The supernatant was adjusted to pH 4.5 with glacial acetic acid in the cold, stirred for 30 min and centrifuged. The sediment was re-dissolved in borate buffer, dialyzed extensively against distilled water and freeze-dried (legumins fraction). The supernatant was also extensively dialyzed against distilled water and centrifuged. The new sediment was freeze-dried (vicilins fraction), and the supernatant was treated with (NH_4_)_2_SO_4_, stirred for 2 h in the cold, and centrifuged. This last sediment (albumins fraction) was dialyzed extensively against distilled water and freeze-dried.

### 2.3. Production of Pea Proteins Hydrolysates

Lactalbumin (Sigma) and pea seed proteins (albumins, legumins and vicilins) were subjected to a gastrointestinal in vitro digestion procedure to obtain protein hydrolysates, namely lactalbumin hydrolysate (Lactalb. H) and pea albumins, legumins and vicilins hydrolysates (PAH, PLH and PVH, respectively). The procedure followed was as previously described [30,31]. Briefly, pea proteins were suspended in 120 mM NaCl. The pH was then adjusted to pH 10.0 with 0.1 M NaOH, and samples were allowed to stand at room temperature for 15 min. Aliquots were taken from each tube for protein analysis at time 0. The pH was then adjusted to pH 2.0 with 5 N HCl and the volume brought to 30 mL with 120 mM NaCl solution. For the gastric digestion, 0.3 mL of pepsin solution (5 mg in 2.5 mL of 0.1 N HCl) was added to each sample, and tubes were gently shaken at 37 °C for 1 h. For the intestinal digestion step, the pH was raised to 6.0 with 1 M NaHCO_3_, and a pancreatin-bile salts mixture was added. The pH was re-adjusted to 7.5 with 1 N NaOH and the volume brought to 45 mL with 120 mM NaCl. Controls containing only the digestive enzymes in buffered solution were included in the assay. Intestinal digestion of proteins was carried out at 37 °C for 2 h. After protein digestion was completed, enzymes were inactivated by heating at 85 °C for 5 min in a water bath, and samples were freeze dried.

### 2.4. Cell Culture and Viability Assay

3T3-L1 preadipocytes subline (derived from 3T3 fibroblast of albino Swiss mice embryo) supplied by the Stem Cell Bank at the CIC (Science Instrumentation Centre, University of Granada, Granada, Spain) was cultured in growth medium composed of DMEM high glucose supplemented with 10% fetal bovine serum, 2 mM glutamine, 50 U/mL penicillin, and 50 µg/mL streptomycin, at 37 °C in 5% CO_2_. Cells were passaged at preconfluent densities using a solution of 0.05% trypsin and 0.5 mM EDTA. To induce differentiation, 3T3-L1 pre-adipocytes were cultured in growth medium until they reached confluence. After 48 h, the cells were incubated in differentiation medium (DM) which was the growth medium supplemented with 10 µg/mL insulin, 1 µM dexamethasone, and 0.5 mM 1-methyl-3-isobutylxanthine (IBMX) in the growth medium with or without 0.5 mg/mL (non cytotoxic concentration) of PAH, PLH or PVH and/or 1 µM rosiglitazone, which was added to increase adipogenesis rate through PPARγ activation. After 48 h, DM medium was changed to post-DM medium (fresh growth medium containing 5 µg/mL insulin with or without PAH, PLH or PVH), and a fresh post-DM medium was supplied every 2 days. 3T3-L1 cells not induced to differentiate were used as pre-adipocytes.

The HCT116 cell line was supplied by the Stem Cell Bank at the CIC (Science Instrumentation Centre, University of Granada, Granada, Spain). Cells were maintained in 12-well cell culture plates using DMEM supplemented with 10% fetal bovine serum, 50 units/mL penicillin, and 50 μg/mL streptomycin at 37 °C in a humidified environment containing 95% air and 5% CO_2_. After confluence, cells were treated with increasing concentrations (0.5–10 mg/mL) of PVH for 48 h. The untreated cells were used as controls. Cell viability was determined by the Neutral Red (NR) Assay [32]. The supernatant of each well was discarded and 1 mL of NR solution (final concentration of 50 μg/mL in DMEM) was added to each well and incubated at 37 °C for 2 h. The NR solution was then aspirated and cells were fixed by immersion in a solution of 0.5% formaldehyde/6.5 mM CaCl_2_ and washed 2 times by immersion in PBS. NR dye was extracted by adding 1 mL per well of a solution of 50% ethanol/1% acetic acid glacial. After incubation for 24 h at 4 °C, plates were agitated (30 min, room temperature). The optical density at 550 nm of wells containing treated and untreated cells was measured by using a spectrophotometer.

### 2.5. 2-deoxy-D-glucose Uptake and Lipid Accumulation Assay

The 3T3-L1 cell line, once differentiated to adipocytes, was used as an in vitro model to study glucose metabolism. This assay was performed for two reasons: (1) because the adipose tissue plays a relevant role in glucose homeostasis [33,34]; and (2) because the 3T3-L1 cell line, once differentiated to adipocytes, is a suitable in vitro model to study glucose metabolism [35]. Eight days after differentiation induction (see above), 3T3-L1 cells were incubated for 48 h with growth medium in the presence or absence of 0.5 mg/mL of Lactalb. H, PAH, PLH or PVH. Lactalb. H was included for comparison with a protein of animal origin, and to also evaluate the effect of intact proteins. After incubation, the supernatant was aspirated and each well was washed twice with PBS. The cells were then deprived of serum by incubation for 3 h in DMEM, washed and incubated during 1 h with or without 100 nM insulin and with or without 0.5 mg/mL of Lactalb. H, PAH, PLH or PVH for 1 h in 2 mL of KRH buffer (10 mM HEPES-NaOH, pH 7.4), 118 mM NaCl, 4.7 mM KCl, 1.2 mM MgSO_4_, 2.5 mM CaCl_2_, 1 mM glucose, 2 mM sodium pyruvate and 0.1% fatty acid-free bovine serum albumin BSA). The medium was recovered, centrifuged 5 min at maximum speed and the supernatant used for glucose determination with the D-glucose-HK assay kit (Megazyme) according to the manufacturer’s recommendations.

### 2.6. Cell Differentiation-Oil Red Staining

Eight days after differentiation induction (see above), the cells were washed with PBS and fixed with 10% formaldehyde/PBS at room temperature for 1 h. Each well was washed three times with PBS and left to dry overnight. Cells were stained with 1 mL per well of Oil Red Solution (0.5% Oil Red O-isopropyl alcohol/H_2_O (3:2, *v/v*)) for 2 h at room temperature. Each well was then washed four times with distilled water and left to dry. After staining, Oil Red O was extracted from cells by adding 1 mL of isopropyl alcohol, and absorbance was measured at 510 nm. Relative lipid accumulation (RLA (%)) was expressed as:

RLA (%) = ((Ac − At)/Ac) × 100

where Ac denotes the absorbance of control cell culture and At denotes the absorbance of treated cells.

### 2.7. RNA Preparation and Real Time rt-PCR Assay

Total RNA was extracted from 3T3-L1 cells by using the RNeasy Mini kit (Qiagen, Manchester, UK) according to the manufacturer’s protocol. RNA quality and quantity were determined by automated electrophoresis (Experion™ Automated Electrophoresis System, Bio-Rad, Madrid, Spain) and spectrophotometry (Nanodrop ND-1000, Nanodrop Labtech, Palaiseau, France). Of the total RNA obtained, 1 μg was used for reverse-transcription which was carried out following the QuantiTect Reverse Transcription Kit (Qiagen, Manchester, UK) protocol. To quantify mRNA expression of adipocyte fatty acid-binding protein (aP2), pre-adipocyte factor-1 (Pref-1), adiponectin, Glut-4 and PPARγ genes, qPCR was performed in an IQ5-Biorad system (Bio-Rad Laboratories Ltd., Mississauga, ON, Canada) by using the iQ™ SYBR^®^ Green Supermix kit (Bio-Rad, Laboratories Ltd., Mississauga, ON, Canada). The oligonucleotide primers used were as in Goto et al. [27] (Table 1). Melting curves were systematically monitored to ensure that only one unique fragment was amplified. Amplification data were expressed as change in cycle threshold (ΔCt) and calculated by using the expression: ΔCt = Cycle threshold of Gene of Interest—Cycle threshold of 36B4. A smaller ΔCt value equates to more abundant transcript.

### 2.8. PPARγ Ligand Activity

HCT116 cells were transiently transfected with the plasmid PPREx3-tK-luc (kindly provided by Dr. Ronald Evans, SIBS, San Diego, CA, USA) and with a PPARγ expressing plasmid using the Fugene-6 transfection reagent (Roche Applied Science, Madrid, Spain). We used HCT116 cells in this case instead of 3T3-L1 preadipocytes because we obtained a better transfection efficiency with HCT116 cells, and also because we preferred to use the same model previously used [36] with the same plasmid (PPREx3-tK-luc). To normalize the transfection efficiency, HCT116 cells were co-transfected with 0.1 μg/well pRL-null renilla luciferase. Different concentrations of PVH were added 12 h after transfection, and cells were cultured for 24 h. Luciferase activities were measured by using the Dual-Luciferase Reporter Assay System (Promega, Madrid, Spain) after lysing of the cells and according to the manufacturer’s instructions. Relative luciferase activities were expressed as fold-activation relative to the control (without samples). Relative light units were examined in triplicate and normalized to renilla.

### 2.9. Partial Characterization of PV and PVH

In view of the preceding results, we thought it convenient to characterize PVH. Thus, pea vicilin (PV) and its enzymatic hydrolysate (PVH) were monitored by size exclusion chromatography (SEC). PV or PVH (10 mg dissolved in 3.0 mL 50 mM Tris-HCl pH 7.5) were filtered through a 0.22 µm filter and 2 mL samples were loaded onto a HiPrep 26/60 Sephacryl S-100 HR column in 50 mM Tris-HCl, pH 7.5 (flow rate of 0.3 mL per min). Three replicates per sample were analyzed. Size calibration of the column separation was based on three sets of standards (set 1: blue dextran, ovalbumin (48.1 kDa), ribonuclease A (15.6 kDa); set 2: blue dextran, bovine serum albumin (63.5 kDa), chymotrypsinogen A (20.4 kDa); set 3: bovine serum albumin, aprotinin (6.5 kDa)) each in 2 mL buffer.

Denaturing gel analyses of pea proteins were carried out by using gradient 4–12% Bis-Tris pre-cast gels (Invitrogen, Madrid, Spain) with 2-*N*-morpholine-ethane sulphonic acid (NuPAGE MES, Invitrogen) as running buffer according to the manufacturer’s instructions. Samples were reduced, immediately before loading, with DTT and NuPAGE antioxidant added to the upper buffer chamber to prevent re-oxidation of reduced proteins during electrophoresis. Gels were stained using Colloidal Blue (Expedeon, Harston, UK). For accurate molecular weight estimation, the unstained protein standard Mark12TM (Invitrogen, LC5677, Spain) was included in the gel with proteins in the range of 2.5 to 200 kDa.

### 2.10. Statistical Analysis

All assays were run in triplicate. The results were expressed as means and SD or SEM. The statistical significance of differences between 2 groups was evaluated by using the unpaired Student’s *t*-test or ANOVA and the Tukey HSD test to determine the differences between mean values of multiple groups. Differences were considered significant for *p* < 0.05 or lower. Discriminant Analysis was carried out by using XLSTAT (2017) [37].

## 3. Results

### 3.1. PVH Enhanced Lipid Accumulation in 3T3-L1 Cells

**Figure 1:**
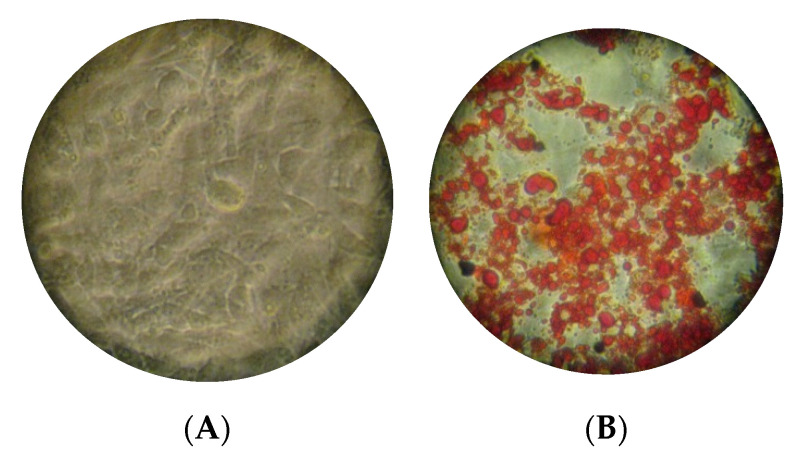
Oil-Red-O stained 3T3-L1 adipocytes not treated (**A**) or treated (**B**) with pea vicilin hydrolysates (PVH) in the medium. Scale bars in panels represent 250 μm.

**Figure 2:**
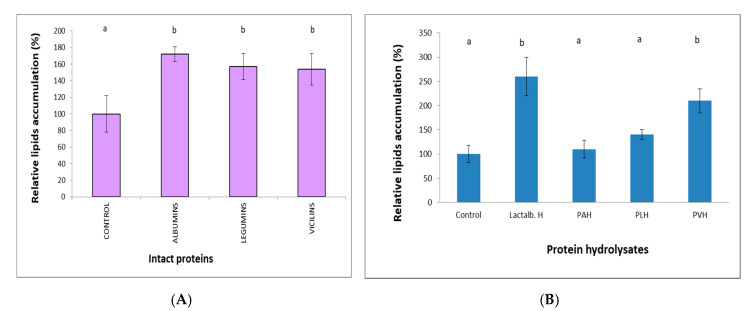
Relative lipid accumulation (% of Oil Red staining) eight days after differentiation induction in 3T3-L1 cells grown in a medium containing intact proteins (1 mg/mL) (**A**) or lactalbumin hydrolysate (Lactalb. H) and pea albumin, legumin or vicilin hydrolysates (PAH, PLH or PVH, respectively) (0.5 mg/mL) (**B**) in the presence of rosiglitazone. Data are the means of 12 independent experiments with their SD in bars. ^a,b^ Bars with different superscript letters indicate significant differences (*p* < 0.01) in relative lipid accumulation with respect to controls.

Adipogenesis was evaluated by Oil Red O staining (Figure 1). Quantification of the extracted Oil Red O dye (Figure 2) revealed that lipid accumulation in 3T3-L1 cells was significantly (*p* < 0.01) increased with the addition of Lactalb. H or PVH, but not with PAH or PLH. Non hydrolyzed proteins (1 mg/mL) (Figure 2A) gave rise to a significant (*p* < 0.01) but very limited effect on relative lipids accumulation, which was not different between proteins. Both Lactalb. H and PVH treatment, but not PAH or PLH, enhanced lipid accumulation (*p* < 0.01) in 3T3-L1 cells during adipocyte differentiation (Figure 2B). Work with PAH was not pursued from here due to the lack of effect, whereas we kept PLH in the assays because legumins belong to the same type of proteins (storage globulins) as vicilins.

### 3.2. PVH Enhanced Gucose Uptake in 3T3-L1 Cells

**Figure 3:**
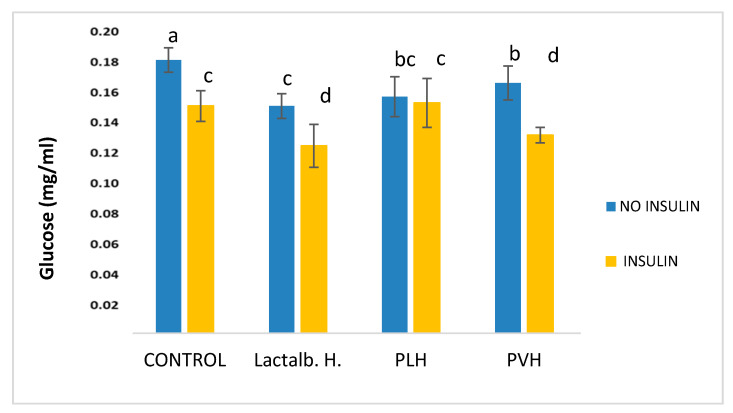
Amounts of glucose determined in the culture medium of differentiated 3T3-L1 cells incubated for 48 h with growth medium containing 0.5 mg/mL of Lactalb. H, PLH and PVH in the absence or presence of insulin. Data are the means of 12 independent experiments with their SD in bars. ^a,b,c,d^ Bars with different superscript letters indicate significant differences (*p* < 0.01) in glucose consumption with respect to control with no hydrolysate added.

The results of the amounts of glucose determined in the culture medium of differentiated 3T3-L1 cells treated with non-cytotoxic 0.5 mg/mL of Lactalb. H, PLH and PVH in the absence or presence of insulin are shown in Figure 3. All hydrolysates induced a reduction (*p* < 0.05) in the amounts of glucose in the medium in the absence of insulin. As expected, insulin addition induced a significant (*p* < 0.05) decrease in the amounts of glucose in the medium in the control cells (in the absence of any hydrolysate). Furthermore, the addition of either Lactalb. H or PVH to the medium in the presence of insulin resulted in a further decrease in the amounts of glucose with respect to cells treated with Lactalb. H or PVH in the absence of insulin. This effect was not found after PLH addition. Linked with the glucose metabolism we determined the effects on the expression of glucose transporter 4 (GLUT4) that encodes the insulin-sensitive glucose transporter induced during adipocyte differentiation (see below).

### 3.3. PVH Increased the Expression Levels of Marker Genes in 3T3-L1 Adipocytes

**Figure 4:**
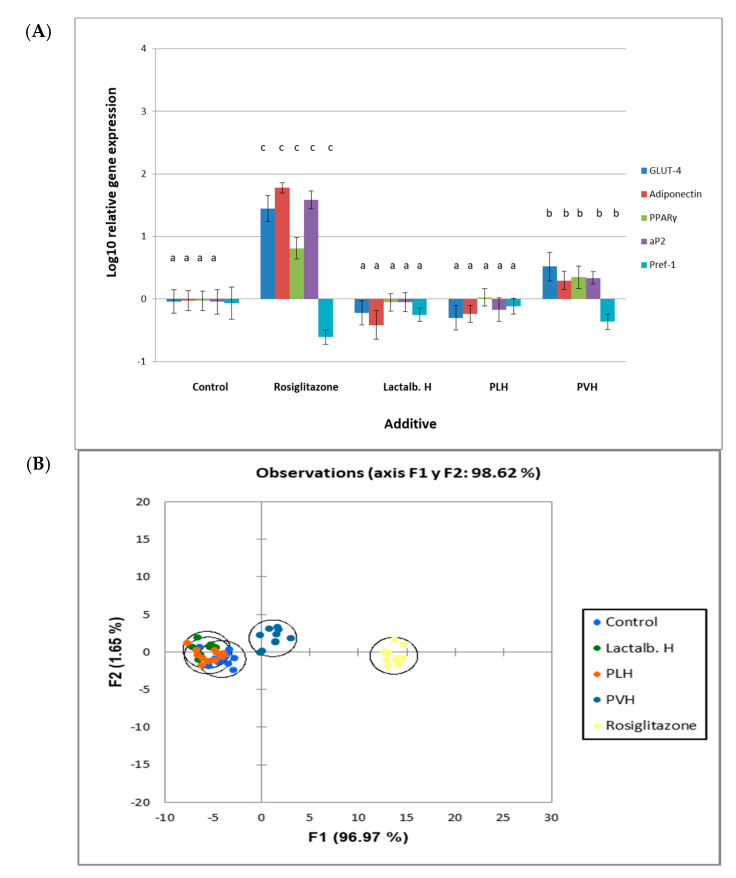
Relative gene expression in 3T3-L1 cell cultures either non treated (control) or treated with rosiglitazone, lactalbumin hydrolysate (Lactalb. H), or pea legumin or vicilin hydrolysates (PLH or PVH, respectively). (**A**) Relative gene (glucose transporter 4 (GLUT4), adiponectin, peroxisome proliferator-activated receptor γ (PPARγ), adipocyte fatty acid-binding protein (aP2), pre-adipocyte factor-1 (Pref-1)) expression in 3T3-L1 cell cultures. Expression analysis was carried out by rt-PCR. Y axis represents log_10_ fold change. Data are means of 12 independent experiments with their SD in bars. ^a,b,c^ Bars with different superscript letters denote significant differences (*p* < 0.01). (**B**) Discriminant Analysis on data of gene expression results in 3T3-L1 cell cultures either non treated (control) or treated with rosiglitazone or hydrolysates (Lactalb. H, PLH, PVH). Differences between rosiglitazone or PVH with controls were significant (*p* < 0.01).

Since PVH affected lipid accumulation and glucose uptake we wanted to investigate whether treatment with this fraction may induce alterations in the mRNA expression levels of adipocyte and preadipocyte marker genes. The expression levels (Figure 4A) of glucose transporter 4 (GLUT4), adiponectin, peroxisome proliferator-activated receptor (PPARγ) and adipocyte fatty acid-binding protein (aP2) were up-regulated after treatment with rosiglitazone or PVH, whereas the preadipocyte factor-1 (Pref-1) marker gene was down-regulated. Lactalb. H or PLH had no significant effect on the expression levels of these marker genes. Discriminant analysis (Figure 4B) showed that the differences in the overall effect on gene expression in 3T3-L1 cell cultures between rosiglitazone or PVH with controls or other proteins were significant (*p* < 0.01).

### 3.4. PVH Treatment Exhibited PPARγ Ligand Activity

**Figure 5:**
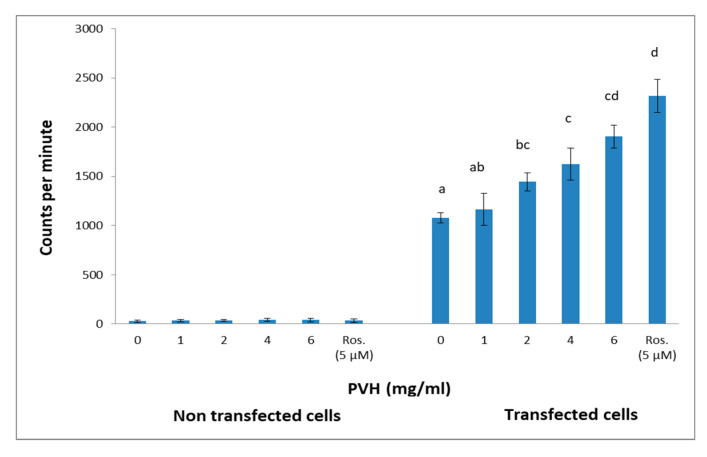
Relative luciferase activities in non-transfected or transfected HCT116 cells expressed as fold-activation relative to the control (without protein hydrolysates), and normalized to renilla. Different concentrations of PVH were added 12 h after transfection and cells were cultured for 24 h. A treatment with rosiglitazone (5 μM) was included for comparison. Data are the means of 12 independent experiments with their SD in bars. ^a,b,c,d^ Bars with different superscript letters indicate significant differences (*p* < 0.01) with respect to control with no rosiglitazone.

**Figure 6:**
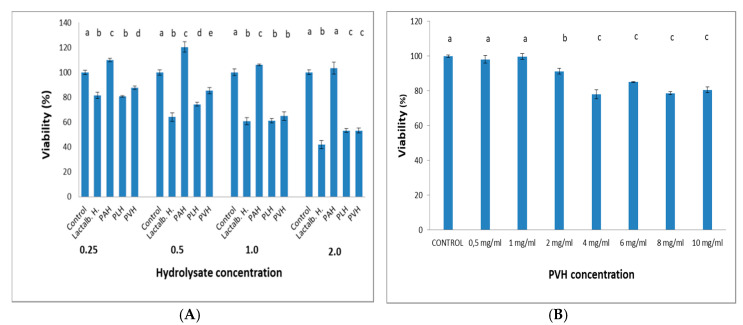
(**A**) Viability of 3T3-L1 cells for 48 h in the presence of increasing concentrations (mg/mL) of Lactalb. H, PAH, PLH or PVH, and (**B**) viability of PVH in HCT116 cells. Data are means of 12 independent experiments with their SD in bars. ^a,b,c,d,e^ Bars with different superscript letters indicate significant differences (*p* < 0.05) with respect to control.

PPARγ belongs to a group of nuclear receptor proteins of ligand-inducible transcription factors regulating the expression of many genes involved in carbohydrate and lipid metabolism. Some natural products have been reported to act as ligands for PPARγ [27,38]. Using a luciferase system, we were interested in testing whether or not PVH served as a ligand for PPARγ. Rosiglitazone, an efficient sensitizer drug, markedly activated the expression of the luciferase reporter gene. The luciferase assay revealed that the reporter gene activity was significantly increased in a dose dependent manner for amounts of PVH > 2 mg/mL (Figure 5) in transfected HCT116 cells. This activity was not different from rosiglitazone (5 μM) when cells were incubated with 6 mg PVH/mL in the medium. This effect was not found with non-transfected HCT116 cells (Figure 5). None of the concentrations of PVH used in this assay were cytotoxic, as shown in Figure 6A (3T3-L1 cells) and Figure 6B (HCT116 cells).

### 3.5. Partial Characterization of PV and PVH

**Figure 7:**
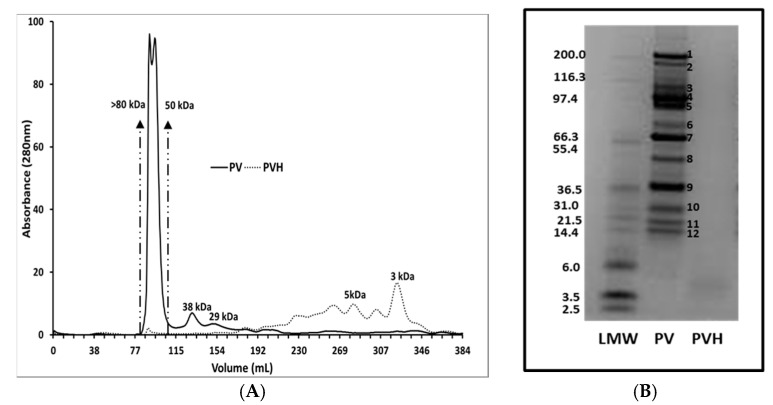
(**A**) Electrophoretic bands from vicilin fraction were previously identified in Rubio et al. [14]. (**B**) Bands 1–3 were identified as convicilin; bands 4, 5, 8 and 9 as vicilin; band 6 as a 38 mixture of vicilin and convicilin; bands 7, 10, 11, 12 as provicilin.

Due to the results obtained with PVH, we thought it was necessary to partly characterize the intact pea protein fractions and its digests. As reported in our previous work [14], putative identification by mass peptide fingerprinting of pea vicilin electrophoretic bands confirmed the lack of cross-contamination with other major pea protein fractions, like albumins and legumin. The amino acids composition of the pea protein fractions was provided in that work [14]. Intact vicilins and their derived protein hydrolysate were analyzed by SEC and Coomassie-stained SDS-PAGE. Thus, SEC profile of intact vicilin (Figure 7A) showed the presence of a major chromatographic double peak that comprises a wide range of molecular weight proteins (50–80 kDa) and other minor chromatographic peaks corresponding to smaller proteins. The chromatographic profile of digested vicilin proteins revealed that PVH was mainly composed of peptides of molecular masses lower than 6 kDa. Although similar amounts of intact and digested vicilin were loaded onto SEC columns, it is necessary to consider that absorption at 280 nm of intact proteins and derived peptides might have varying absorption characteristics and chromatograms need to be considered only for qualitative purposes. In agreement with our previous paper [14], the vicilin fraction consisted of a complex mixture of vicilin and convicilin polypeptides. Electrophoretic analysis of the vicilin fraction and further putative identification by mass peptide fingerprinting of their major components demonstrated a lack of cross-contamination in the vicilin fraction from albumin and legumin components. Convicilin polypeptides were reported as those of lower relative mobility (≥70 Da) while vicilin mostly consisted of polypeptides lower than 70 kDa derived from limited post-translational processing (Figure 7B). For comparative purposes, the amount of intact and digested vicilin loaded onto Bis-Tris gels having an optimal resolution up to 2.5 kDa was similar. Hydrolysis of the vicilin fraction caused the disappearance of electrophoretic bands (Figure 7B). In vitro digestibility of this fraction is extremely elevated [14], with values significantly higher (88%) when compared with both albumin and legumin fractions (41 and 63%, respectively).

## 4. Discussion

It has been reported [39] that rats fed a high-fat soy protein diet showed a significantly smaller area and a higher adipocyte number than those fed a high-fat casein diet. This suggests that soy protein consumption may affect adipocyte differentiation in vivo, although the mechanisms implicated in the effects of soy (or other legume) proteins on adipocyte differentiation are not fully understood. In an attempt to establish the molecular mechanisms involved in those effects on adipocytes, Goto et al. [27] showed that a soy protein hydrolysate induced changes in a number of key markers of glucose absorption and cell differentiation in adipocytes (3T3-L1) cultures. Accordingly, taking into account the effects previously found in vitro and in vivo with other legume proteins as explained above, we decided to study more deeply the effects of pea albumins and major globulin storage protein fraction (legumins and vicilins) hydrolysates on 3T3-L1 cells’ metabolism in vitro.

It is already known that obesity causes excess fat accumulation in various tissues such as white adipose tissue (WAT), and WAT dysfunction has been shown to play a key role in the development of obesity, obesity associated diseases, and accompanying disorders such as insulin resistance [40]. Regulation of adipocyte differentiation is closely linked to obesity and insulin resistance, and the number of adipocytes is thought to increase because of the proliferation of pre-adipocytes and subsequent differentiation into mature adipocytes [41]. In the current work, we have shown that PVH treatment of 3T3-L1 cells induced a significant effect on differentiation (Figure 2) (measured as Oil Red staining, Figure 1). Additionally, Lactalb. H and PVH decreased the amount of glucose in the medium in the presence of insulin (Figure 3), which indicates increased glucose consumption by the cells when these pea protein hydrolysates were added to the medium, an effect probably mediated by the increased expression of GLUT4 (Figure 4A) (see below). In addition, culturing of 3T3-L1 cells in the presence of PAH or PLH did not induce any significant effect on differentiation, while the PVH gave rise to a significant increase with respect to non-treated cells. Interestingly, also Lactalb. H was found to enhance cell differentiation in the presence of rosiglitazone (Figure 2B). These results indicate that PVH treatment enhances glucose uptake, lipid accumulation and adipocyte differentiation in 3T3-L1 cells.

We then evaluated the expression levels of a number of genes implicated in adipocytes metabolism. Thus, we found that GLUT4, adiponectin, aP2 and PPARγ were upregulated, while Pref-1 was downregulated, with both rosiglitazone and PVH, while there was no effect with Lactalb. H or PLH (Figure 4A). The effect of rosiglitazone was however much stronger than that found with PVH. The increase by PVH in the mRNA expression levels of aP2, a well-known adipocyte marker gene, and the decrease in those of Pref-1, a well-known pre-adipocyte marker gene, is in line with the results on cell differentiation already indicated. In addition, GLUT4 (an insulin-sensitive glucose transporter), adiponectin (an antiatherosclerotic and antidiabetic hormone specifically derived from adipocytes) and PPARγ (a key regulator of adipocyte differentiation) [40,42] were all upregulated by PVH and rosiglitazone, which adds more to the antidiabetic, antiatherosclerotic and differentiation enhancing attributes of PVH.

These results are in line with previous results by Goto et al. [27] with soybean protein hydrolysates. However, the mechanism underlying the up-regulation of PPARγ expression levels in PVH-treated 3T3-L1 cells is unknown. In the case of the soybean hydrolysate, Goto et al. [27] suggested the activation of extracellular signal-regulated kinase (ERK)-C/EBPβ pathway, which stimulates PPARγ transcription, as soy peptides have been shown to induce phosphorylation of ERK in human adipose tissue-derived mesenchymal stem cells. No such mechanism has been described so far for pea storage proteins, although other biological activities such as antioxidant, antiinflammatory and immunomodulating properties theoretically attributed to lunasin and lunasin-like peptides have been described for pea protein hydrolysates [43]. The results on mRNA expression are summarized when analyzed using Discriminate Analysis. As shown in Figure 4B, the effects of rosiglitazone and PVH were in the same line and different from those obtained with the other proteins’ hydrolysates, albeit rosiglitazone’s effect was much stronger.

As for the PPARγ ligand activity (Figure 5), this is the first time to the best of our knowledge that such an effect is described for a legume protein hydrolysate. Peroxisome proliferator-activated receptors (PPARs) are ligand dependent transcription factors which are regarded as key regulators of fatty acid and lipoprotein metabolism, glucose homeostasis, cellular proliferation/differentiation and the immune response. The abundant pleiotropic actions of PPARs suggest that PPAR agonists have enormous therapeutic potential, and there is therefore growing interest in the development of cell-specific PPAR agonists and in the improvement of clinical uses for PPAR ligands. PPARs are therefore important targets in the treatment of metabolic disorders such as insulin resistance and type 2 diabetes mellitus. In particular, PPARγ is an essential modulator of cell differentiation and lipid storage in WAT, it contributes to insulin sensitivity, reduces the secretion of inflammatory cytokines, increases the plasma level of adiponectin; PPARγ suppression also reduces GLUT4-mediated glucose uptake, and plays important anti-inflammatory roles in macrophages and other tissues [44]. Of note in the nutritional context, a number of fatty acids such as polyunsaturated fatty acids, and other nutrients such as glutamine, spicy food or flavonoids are also able to activate PPARγ, which opens the way for potential roles of dietary compounds in modulating intestinal inflammation through PPARγ [45]. Another recently described active natural edible product is amorfrutins (a family of isoprenoid-substituted benzoic acid derivatives from edible parts of the legumes *Glycyrrhiza foetida* and *Amorpha fruticose*) which are able to bind and activate PPARγ. This activation results in selective gene expression and physiological profiles such as improvement of insulin resistance and other metabolic and inflammatory parameters without a concomitant increase in fat storage or other unwanted side effects such as hepatotoxicity [46]. Our current results point to the possibility of considering PVH as a new potential PPARγ agonist since PVH not only increased its expression level but also showed ligand activity. With our current information, it is difficult to speculate on the possible mechanism of PVH ligand activity. A number of biological activities linked to non-communicable diseases such as obesity and type-2 diabetes have been recently reported for legume-derived peptides [47]. Although pea globulins-derived bioactive peptides have been reported, activities were linked to the inhibition of Angiotensin Converting Enzyme [48]. To our knowledge, only a single study [49] has reported an effect of amino acids on PPARγ activity, where authors suggested that glutamine is also a ligand for PPARγ. Pea globulins, and particularly vicilins are rich in glutamate [14], and legume protein glutamate is readily absorbed both in vivo and in vitro [50]. Therefore, either free glutamine derived from glutamate or a glutamate-containing peptide might be acting as a PPARγ agonist in PVH. Finally, PPARγ suppression reduces GLUT4–mediated glucose uptake and lipogenesis in adipose tissue [44], which may also explain the effects found here on glucose uptake and lipids accumulation.

Although more extensive work has been undertaken with some vegetable products such as soy isoflavones (genistein, daidzein, etc.) in adipocyte differentiation, not much information exists on the effects of specific legume proteins/peptides. Some recent work has been reported with synthetic milk peptides [51] or the soy-derived peptide Phe-Leu-Val [52]. While some of the effects described for those peptides are similar to those reported here, no such effects have been described previously to our knowledge for any legume protein apart from soybean [27]. The two main differences in the current work compared to that previous work were, firstly that we used a well-defined pea protein fraction (vicilins) [14] instead of a whole seed protein hydrolysate; and secondly that we found an effect of PVH on PPARγ ligand activity, which was not reported with the soybean protein hydrolysate. The fact that the effects were found with the vicilins fraction has the advantage that it significantly confines the search for a hypothetic active peptide(s) to pea seed proteins, and in particular to those derived from a gastrointestinal digestion. The hydrolysis process here used was quite effective as the PVH obtained contained no evident electrophoretic bands resistant to gastrointestinal digestion, and the remaining peptides were under 6 kDa, as observed by SEC (Figure 6). The description of such specific peptide(s) (work under way) would open the way for mechanistic explanations on the activity of legume proteins on lipid metabolism, and would also ease the design of other active molecules.

## 5. Conclusions

In summary, PVH, a protein hydrolysate obtained from a well-defined pea vicilin protein fraction after a simulated gastrointestinal digestion, modulated the mRNA expression levels of markers of differentiation and glucose uptake and metabolism in 3T3-L1 adipocytes. In particular, PVH was found to up-regulate the expression levels of PPARγ and exhibited PPARγ ligand activity. These findings show that PVH stimulates adipocyte differentiation via, at least in part, the up-regulation of PPARγ expression levels and ligand activity. These effects of PVH might be relevant in the context of the health beneficial effects of legume consumption in obesity-associated metabolic disorders.

## Figures and Tables

**Table 1:** Primers for real-time rt-PCR (as in Goto et al. [27]).

Gene	Primer Sequences	Accession No.
Adiponectin	Fw: ACAACCAACAGAATCATTATGACGG	
	Rv: GAAAGCCAGTAAATGTAGAGTCGTTGA	NM_009605.4
Adipocyte fatty acid-binding protein (aP2)	Fw: AAGACAGCTCCTCCTCGAAGGTT	
	Rv: TGACCAAATCCCCATTTACGC	NM_024406.2
Glucose transporter (Glut4)	Fw: CGGATGCTATGGGTCCTTACG	
	Rv: TGAGATCTGGTCAAACGTCCG	NM_009204
Peroxisome proliferator-activated receptor γ (PPARγ)	Fw: GGAGATCTCCAGTGATATCGACCA	
	Rv: ACGGCTTCTACGGATCGAAACT	NM_001127330.1
Pre-adipocyte factor-1 (Pref-1)	Fw: GTGACCCCCAGTATGGATTC	
	Rv: AGGGAGAACCATTGATCACG	NR_033813.1
36B4	Fw: TGTGTGTCTGCAGATCGGGTAC	
	Rv: CTTTGGCGGGATTAGTCGAAG	NM_007475.5

## Abbreviations

aP2	Adipocyte fatty acid-binding protein 2
GLUT4	Insulin-responsive glucose transporter 4
Lactalb. H	Lactalbumin hydrolysate
PAH	Pea albumin hydrolysate
PLH	Pea legumin hydrolysate
PPARγ	Peroxisome proliferator-activated receptor γ
Pref-1	Preadipocyte factor-1
PVH	Pea vicilin hydrolysate
SEC	Size-exclusion chromatography
WAT	White adipose tissue
